# The Effect of Hybrosome (Umbilical Cord Blood Exosome–Liposome Hybrid Vesicles) on Human Dermal Cells In Vitro

**DOI:** 10.1093/asjof/ojad039

**Published:** 2023-04-25

**Authors:** Polen Koçak, Naz Unsal, Serli Canikyan, Yaren Kul, Steven R Cohen, Tunç Tiryaki, Diane Duncan, Kai-Uwe Schlaudraff, Benjamin Ascher, Teodor Eren Tiryaki

## Abstract

**Background:**

Wound healing is a process that involves multiple physiological steps, and despite the availability of various wound treatment methods, their effectiveness is still limited due to several factors, including cost, efficiency, patient-specific requirements, and side effects. In recent years, nanovesicles called exosomes have gained increasing attention as a potential wound care solution due to their unique cargo components which enable cell-to-cell communication and regulate various biological processes. Umbilical cord blood plasma (UCBP) exosomes have shown promise in triggering beneficial signaling pathways that aid in cell proliferation and wound healing. However, there is still very limited information about the wound-healing effect of UCBP exosomes in the literature.

**Objectives:**

The primary objective of this study was to investigate the “hybrosome” technology generated with calf UCBP-derived exosome–liposome combination.

**Methods:**

The authors developed hybrosome technology by fusing cord blood exosome membranes with liposomes. Nanovesicle characterization, cell proliferation assay, wound-healing scratch assay, immunohistochemistry analysis, anti-inflammation assay, real-time polymerase chain reaction (RT-PCR), enzyme-linked immunosorbent assay, and cellular uptake studies were performed using the novel hybrid exosomes.

**Results:**

Experimental results showed that hybrosome increases cell proliferation and migration by 40% to 50%, depending on the dose, and induces an anti-inflammatory effect on different cell lines as well as increased wound healing–related gene expression levels in dermal cells in vitro. All in all, this research widens the scope of wound-healing therapeutics to the novel hybrosome technology.

**Conclusions:**

UCBP-based applications have the potential for wound treatments and are promising in the development of novel therapies. This study shows that hybrosomes have outstanding abilities in wound healing using in vitro approaches.

**Level of Evidence: 3:**

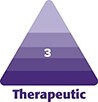

The wound-healing process in plastic surgery is an important aspect of the procedure. After surgery, the body goes through a complex series of events to repair and regenerate damaged tissues. The wound-healing process can be divided into 4 phases: homeostasis, the inflammatory phase, the proliferative phase, and the remodeling phase.^1,2^ A group of regulatory molecules such as platelets, endothelial cells, inflammatory cells, cytokines, and growth factors play crucial roles in these processes.^1,3^ The initial phase of wound healing is known as homeostasis, which consists of coagulation and fibrin clot formation. The inflammatory phase follows homeostasis and constituents as the main defense of the body against pathogenic infections.^4,5^ After inflammation, dermal and epidermal cells migrate through the wound site and regulate the proliferation phase. In the final remodeling stage of healing, the matrix accumulated in the wound area is reshaped by fibroblasts, and the wound becomes completely epithelialized over time.^2,4^

Cell-to-cell communication is essential to ensure optimal coordination between different cell types in tissues and to maintain tissue homeostasis and organism integrity.^6,7^ Multicellular organisms secrete chemical signals that can travel long distances and generally communicate through such signaling molecules and receptors.^7,8^ In the wound-healing process, direct intercellular communication proceeds through gap junctional intercellular communications. Gap junctional intercellular communication between mast cells and fibroblasts is vital in the remodeling phase. At the same time, recent studies have revealed that interaction between cells can also occur through extracellular vesicles such as exosomes.^6^

Exosomes are the most well-known extracellular vesicle type, with sizes ranging from 20 to 130 nm.^9^ Exosomes can be found in several sources, including stem cells, body fluids, immune cells, epithelial and endothelial cells, and cancer cells, and are subsequently secreted by the parent cell.^10^ They have a diverse biological content, consisting of micro messenger RNA (mRNA), mRNA, DNA, lipids, and proteins, and they play a crucial role in intercellular signaling and genetic material transfer.^8^ Although exosomes were considered cellular waste when they were first discovered, they have proved to play an important role in cell-to-cell communication.^11^ Nowadays, exosomes are used as smart therapeutic agents especially in wound healing because of their role in cell–cell communication and their capacity to diffuse through the tissue to reach target cells. Exosomes derived from various cell types, including stem cells, immune cells, plasma, and mesenchymal cells, have been shown to have beneficial effects on tissue repair and wound healing. These exosomes can promote cell proliferation, angiogenesis (the formation of new blood vessels), and anti-inflammatory responses, and they can also enhance tissue regeneration and repair.^12^

Nowadays, umbilical cord blood plasma (UCBP)-based applications are increasingly popular for regenerative medicine and wound repair studies. There are several studies in the literature investigating the effects of UCBP products. For example, exosomes derived from UCBP have shown excellent potential for wound healing due to their properties such as increasing epithelial cell proliferation and migration, reducing wound widths, and promoting angiogenic activities. In addition, animal wound models showed that after UCBP exosome treatment; collagen deposition, new capillary formation, and rapid re-epithelialization were facilitated as well as wound closure with less scar tissue formed through the improvement of regenerative activity.^13^ In conclusion, UCBP-based applications have a huge potential for wound treatments and are promising for the development of novel therapies. Since human cord blood is not an easily obtainable source, studies are using animal-derived cord blood, which may have the most compatible protein structure with humans.^14,15^

In wound-healing studies, liposomes can be used to deliver therapeutic agents directly to the site of the wound, improving the efficiency and effectiveness of the treatment. Liposomes are small, spherical-shaped particles primarily made up of various types of lipids and organized into one or more lipid bilayers.^16^ Since studies with liposomes have been ongoing for more than 50 years, they have become well-established and clinically approved delivery vesicles.^17^ For liposomes to acquire smart targeting capabilities, they must perform surface alterations with natural nanovesicles that possess the targeting skills, such as exosomes. Exosomes, as natural nanovesicles secreted by cells, can target specific cell types. Liposomes, on the other hand, can be engineered to carry specific biomolecules and provide stability to the hybrid vesicles in circulation. At this point, to modify and upgrade the cargo-carrying capacity of liposomes, Sato et al used hybridization technology. In this study, a new-generation hybrid exosome–liposome carrier system was formed by fusing the exosome membranes with the liposome membrane by the freeze-thaw method.^18^ In this system, exosomes allow hybrid vesicles to target specific receptor cell types and have high biocompatibility and low immunogenicity, thereby increasing systemic circulatory stability and therapeutic efficacy of hybrid vesicles. On the contrary, liposomes extend the half-lives of hybrid vesicles in circulation by stabilizing them more than exosomes.^12^ Various nanocarrier systems have been developed with this hybridization method and successful studies have been carried out by utilizing the relevant properties of both liposome and exosome together. Hybrid exosomes have been used to create a variety of targeted therapeutic delivery systems for diseases like pulmonary fibrosis and cancer.^19,20^ In addition, Lin et al developed an exosome–liposome hybrid nanocarrier system for the CRISPR Cas-9 system (CRISPR Therapeutics, South Boston, MA), which is one of the most powerful gene editing tools of the new era.^21^

Moreover, hybrid liposome–exosome delivery systems are a promising approach to wound healing by taking advantage of exosomes and liposomes to target specific cells and accelerate wound healing. The success of exosome–liposome hybrid molecules in wound healing depends on several factors, including the properties of the vesicles, the selection of biomolecules for loading, and the delivery method. For instance, the hybrid vesicles need to be stable and capable of delivering the therapeutic molecules to the wound site efficiently. The biomolecules loaded into the vesicles should have wound-healing properties, such as promoting cell growth and differentiation or reducing inflammation. Additionally, the delivery method should be optimized to ensure that the vesicles can reach the wound site effectively.^22,23^ In this study, we developed an innovative wound treatment approach depending on the enhanced wound-healing capability of calf UCBP exosomes, through a new type of nanoparticle produced by the fusion of exosomes and liposomes called “hybrosomes” and controlled their effects on wound healing.

## METHODS

### Cord Blood Collection

Ethics approval was obtained from Yeditepe University Animal Research Ethical Committee (Approval protocol number: 2020-861) on August 27, 2020. The cord blood of a female calf giving birth whose viral tests have been completed was collected according to the protocol with veterinary permission. Cord blood was transferred to a tube/bag containing 1%-3% anticoagulant and gently shaken by turning it upside down at 90° angle. It was centrifuged for 2 to 6 min at a speed of 2000 to 3000× *g*/min, and 2 phases were formed consisting of blood cells and cell debris in the lower part and blood plasma fragments in the upper part. The plasma fragment was a fluid that is rich in growth factors and consists of large and small proteins as well as exosomes.

### Extracellular Vesicle Isolation

Extracellular vesicle isolation from calf cord blood plasma is started with a centrifuge as a first step. To eliminate any residual cell debris, the supernatant was transferred to a new centrifuge tube and spun at 16,000*×g* for 30 min. The supernatant was transferred into a new centrifuge tube, and a 1:2 ratio of Exo-spin Buffer (#EX06-250; Cell Guidance Systems, Cambridge, UK) was added. After mixing well by inverting the tube, it was incubated for 1 h at 4°C. The tube was mixed well by inverting and incubated for 1 h at 4°C, then centrifuged at 16,000*×g* for 1 h. The mixture was carefully aspirated, and the supernatant was discarded. The exosome containing the pellet was resuspended in phosphate-buffered saline (PBS). The resuspended pellet was placed on the Exo-spin column, centrifuged at 50*×g* for 60 s, and the flow-through was removed. The column was placed in a 1.5 mL microcentrifuge tube and PBS was added. Purified exosomes were obtained by centrifugation at 50*×g* for 60 s and passed through a 0.22 μm pore (#SLGVR33RS; Merck Millipore Co., Billerica, MA) polyvinylidene difluoride membrane filter.

### Preparation of Nanoliposome Vesicles

An egg yolk lecithin (L-α-phosphatidylcholine [egg yolk, Type XVI-E, ≥ 99% (TLC), lyophilized powder, P3556; Sigma-Aldrich, Sydney, Australia]) stock solution of 2% (w/v) was prepared under nitrogen flow to prevent oxidation and then stored in the dark at 4°C. Fresh liposomes were prepared through probe sonication (Q500 Sonicator; QSonica, Newtown, CT) of the egg yolk lecithin solution using a 3.2 mm microtip at 30% amplitude and subjected to a pulse mode for 4 min (5 s on-off alterations).

### Preparation of Hybrid Exosome–Liposome Nanovesicles

To initiate the exosome–liposome nanovesicle hybridization process, equal concentrations of UCBP exosomes and egg yolk lecithin liposomes were incubated at 37°C for 12 h. Fresh hybrosome vesicles were prepared through probe sonication (Q500 Sonicator) concerning described settings on the nanoliposome vesicle preparation section.

### Nanoparticle Tracking Analysis

The Nanosight NS300 (Malvern Instruments, Malvern, UK) with a 488 nm laser was used for hybrosomes, UCBP exosomes, and egg yolk lecithin liposomes measurement to estimate their size distribution. Samples were diluted with particle-free PBS (Gibco, Thermo Fischer Scientific, Waltham, MA) according to the manufacturer's instructions, and video capture and imaging were conducted at 16 camera levels at 30 s × 60 s intervals. The samples were analyzed at a constant flow rate of 50 and a room temperature of 25°C. The concentration and size distribution of the hybrosomes, UCBP exosomes, and egg yolk lecithin liposomes were examined by repeating the procedures 5 times.

### Flow Cytometric Analysis

UCBP exosomes, egg yolk lecithin liposomes, and hybrosomes were attached to 10 µL beads (Invitrogen, Thermo Fischer Scientific) by mixing 30 µg of exosomes in a 10 µL volume of beads for 15 min at room temperature, and the suspension volume was completed to 1 mL by using PBS buffer. The prepared solution was incubated overnight at 4°C in a rotary shaker with agitation. The reaction was then stopped with the application of 100 mM glycine and 2% Bovine serum albumin (BSA) for 30 min at room temperature. Following this, exosome- and hybrosome-linked beads were washed in PBS/2% BSA and centrifuged for 1 min at 14,800*×g*. To block, 10% BSA was applied for 30 min and the centrifugation step at 14,800*×g* was repeated for 1 min. The presence of the CD9, CD63, and CD81 biomarker proteins on the membrane surface of exosome and hybrosome bound to the beads was examined by labeling with CD9 (1:400, Cat# 312105; Biolegend, San Diego, CA), CD63 (1:400; Cat# 353005; Biolegend), and CD81 (1:400; Cat# 349509; Biolegend) antibodies. Subsequently, 5 µL of secondary antibodies were added, and exomes and hybrosomes were evaluated by flow cytometric analysis (Attune NxT Flow Cytometer; Thermo Fisher Scientific).

### Cellular Uptake

UCBP exosomes, egg yolk lecithin liposomes, and hybrosomes were labeled with the green lipid membrane dye PKH67 (Sigma-Aldrich, St Louis, MO), according to the manufacturer's protocol. Exosomes, liposomes, hybrosomes, and PKH67 were diluted separately in 1 mL of diluent C. After the dilution step, all groups were mixed with the staining solution and incubated for 5 min at room temperature. 2 mL of 10% BSA is added to stop the labeling, and the mixture is then filtered through a 100 kDa Amicon filter to remove any unincorporated dye. Subsequently, the washing step was conducted with PBS and then the ultracentrifuge step is applied. Afterward, human dermal fibroblast (HDF) cell line, human keratinocyte cell line (HaCaT), and human umbilical vein endothelial cells (HUVECs) were then incubated separately with 100 µg/mL PKH67-labeled exosomes, liposomes, and hybrosomes for 48 h. Samples were fixed with 4% paraformaldehyde for 30 min and permeabilized with 0.1% Triton X-100 for 30 min at room temperature. Before imaging, Hoechst (#62249; Thermo Fischer Scientific) and DAPI (4′,6-diamidino-2-phenylindole, 1:1000; Sigma-Aldrich, St Louis, MO) dyes were also used to stain the cells and nucleus. After samples were washed 3 times with DPBS, the assessment was conducted by fluorescence microscopy.

### Cell Proliferation Assay

For the cell proliferation analysis, HDF, HaCaT, and HUVEC cell lines were purchased from the American Type Culture Collection. HUVEC and HaCaT cells were cultured in Dulbecco's modified essential medium (DMEM) with a high glucose content (Invitrogen, Thermo Fischer Scientific). At cell culture conditions, HDF cells were grown in low-glucose DMEM supplemented with 10% exosome-depleted fetal bovine serum (FBS) and 1% penicillin–streptomycin–amphotericin mixture (Gibco, Thermo Fischer). Cells were seeded in 96-well culture plates at a concentration of 3 × 10^3^ cells/well. Subsequently, culture plates were treated with hybrosomes separately at concentrations of 30-50-100-200 μg/mL for 24, 48, and 72 h in a humidified incubator (5% CO_2_ in air at 37°C). Cell proliferation was assessed with the WST-1 assay (BioVision, Milpitas, CA), according to the manufacturer’s directions, and absorbance was determined at 540 nm with a spectrophotometer.

### Wound Healing Scratch Assay

HDF cells were seeded onto 6-well culture plates (TPP, Trasadingen, Switzerland) at a concentration of 1 × 10^6^ cells/well. Plates were scratched with the aid of a 1000 μL sterile tip, and cell culture media was changed by adding fresh medium containing 2% exosome-depleted FBS and 100 μg/mL hybrosome. At the end of 24 and 48 h cell culture periods in a humidified incubator, wound-healing properties were assessed by using an inverted microscope with a digital camera (Nikon Eclipse TE200: Nikon, Tokyo, Japan).

### Immunocytochemistry Analysis

HDF cells were seeded onto an 8-well chamber with a cell concentration of 3000 cells per chamber and incubated overnight at cell culture conditions. The control group was treated with a complete DMEM medium, and the sample group was treated with 100 μg/mL hybrosome for 24 h, fixed with 2% (w/v) of paraformaldehyde (Sigma-Aldrich, St Louis, MO) solution for 30 min at room temperature, and washed 3 times for 5 min with PBS-T (PBS with 0.1% Tween 20, Sigma-Aldrich). They were treated with 0.1% (v/v) Triton X-100 (Bio Basic Inc, Ontario, Canada) to permeabilize the cells for 5 min at room temperature and washed with PBS-T. The collagen Type I group was incubated overnight at 4°C with 2% goat serum (Sigma-Aldrich, Munich, Germany) and 0.2% primary antibody against collagen Type I (Abcam, Cambridge, UK); the elastin group was incubated overnight at 4°C with 2% goat serum (Sigma-Aldrich, Munich, Germany) and 0.2% primary antibody against elastin (Abcam). The PBS-T washing step was repeated after incubation. Cells were then incubated with 0.4% goat anti-mouse AlexaFluor-647 antibody (Abcam) for 1 h at 4°C and washed gently. For nuclei examination, cells were stained with 0.1% DAPI solution (4′,6-di-di-amidino-2-phenylindole; AppliChem, Darmstadt, Germany) for 20 min at 4°C and washed with PBS-T. Cells were observed using a confocal laser scanning microscope (LSM800; Carl Zeiss, Oberkochen, Germany). For Type I collagen and elastin quantifications, TIFF images were analyzed with ImageJ software (National Institutes of Health, Bethesda, MD).

### Anti-Inflammation Assay

The hydrogen peroxide (H_2_O_2_) anti-inflammation assay was used to assess the response of hybrosomes under the inflammation. HDF, HaCaT, and HUVEC cells were seeded with a cell density of 5 × 10^3^ cells/well onto 96 well plates. Plates were then treated with 1.0 mM H_2_O_2_, 100 μg/mL hybrosome, and 1.0 mM H_2_O_2_ and 100 μg/mL hybrosome together for 24 h at 37°C. After 24 h treatment, the MTS assay was used for the analysis of cell viability.

### Gene Expression Analysis With Real-Time Polymerase Chain Reaction (RT-PCR)

To monitor wound healing, the collagen Type I, laminin, elastin, MMP1, MMP2, and MM9 primers were designed using Primer-BLAST software from the National Center for Biotechnology (Bethesda, MD). Total RNA was extracted from the 100 μg/mL hybrosome-treated HDF cells with TRIzol (Thermo Fisher Scientific). To reverse transcription of RNA to CDNA, the QuantiTect Reverse Transcription Kit (Qiagen, Hilden, Germany) was used according to the manufacturer's protocol. The QuantiTect SYBR Green PCR kit (Qiagen) was used to examine the mRNA expression of the collagen Type I, laminin, elastin, MMP1, MMP2, and MM9 levels. A reaction mixture consisting of SYBR green PCR mix, universal primer, RNase-DNase-free water, and 500 ng for each sample was prepared following the manufacturer's protocol. Reactions were performed with the iCycler RT-PCR system (Bio-Rad, Hercules, CA). 18S rRNA reference gene was used to make relative quantification, and the standard curve was used to analyze absolute quantification.

### Enzyme-Linked Immunosorbent Assays

Human vascular endothelial growth factor (VEGF), human pro-collagen 1 alpha 1, and human transforming growth factor (TGF)-beta 1 enzyme-linked immunosorbent assays (ELISA kits) were performed to see the effect of hybrosomes on HDF and HUVEC cells to investigate the change in protein expression levels. Briefly, 100 μL of hybrosome was seeded into related antibody-coated 96-well plates included in each kit and incubated for 2.5 h. After incubation, the medium was discarded and then the wells were then incubated with 100 μL of biotin antibodies (1X) at room temperature for 1 h. Wells were decanted and washed 3 times with a 1 × wash buffer, and 100 μL of 1× HRP Avidin solution was applied to wells and incubated at room temperature for 1 h. Wells were aspirated and washed 4 times with a 1 × wash buffer and then incubated with 90 μL of TMB substrate solution at dark and room temperature for 30 min. To stop the reaction, 100 μL of the stop solution was added to the wells. Absorbance values of the wells were evaluated with an ELISA plate reader at 450 nm with 620 nm as the reference wavelength.

### Statistical Analysis

For multiple data comparisons using GraphPad Prism statistical software 5.0 (GraphPad Software, La Jolla, CA), 1-way variance analysis (ANOVA) followed by Tukey's post hoc test was carried out.

## RESULTS

### Fabrication and Characterization of Nanovesicles

Liposomes were obtained from egg yolk lecithin, which is rich in phospholipids, cholesterols, carotenoids, and tocopherols, and exosomes were isolated from umbilical cord blood serum through Exo-spin Exosome Purification Kit as described above. Hybrosomes were produced by membrane fusion of UCBP exosomes and liposomes using the probe-sonication method. Briefly, their formation mechanisms rely on the co-incubation of lipid vesicles at 37°C for 12 h to merge UCBP exosome and liposome molecules.

**Figure 1.**
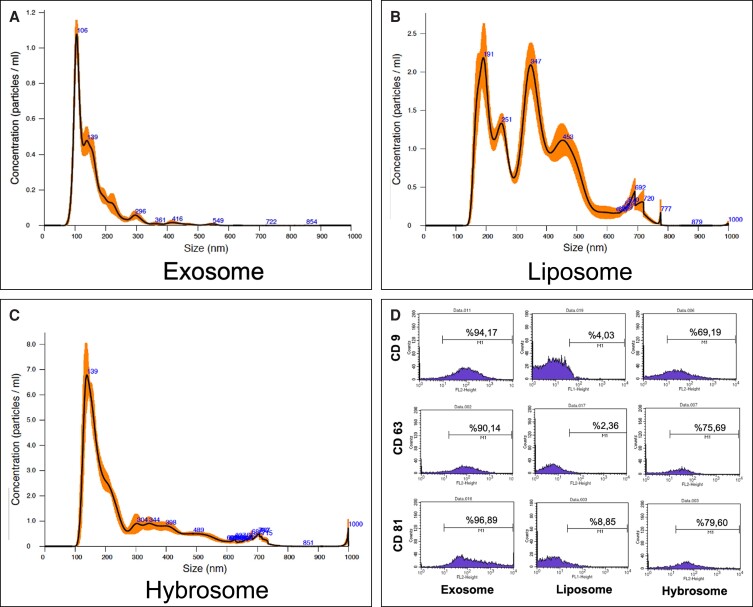
NTA and flow cytometry analysis of the UCBP exosome, liposome, and hybrosome for the characterization. NTA data demonstrated the FTLA concentration/size analysis of the (A) exosome, (B) liposome, and (C) hybrosome. (D) Flow cytometry analysis shows strong bindings between the exosome-specific surface antigens CD9, CD63, and CD81 and the UCBP exosomes as well as hybrid hybrosome nanovesicles (*P* ≤ .05). FTLA, finite track length adjustment; NTA, nanoparticle tracking assay; UCBP, umbilical cord blood plasma.

The size and concentration analysis of UCBP exosomes, egg yolk lecithin liposomes, and hybrosomes were performed by nanoparticle tracking analysis (NTA), which is a gold standard diagnostic test to measure particle size distribution and concentration in biological samples. According to the NTA analysis results, the main particle sizes of the exosome, liposome, and hybrosome molecules range around 106, 191, and 139 nm respectively (Figure 1A-C).

The percentage assessment of exosome-specific CD surface markers on the surfaces of the UCBP exosomes, liposomes, and hybrosomes was performed through a flow cytometer (Figure 1D). Exosomes, liposomes, and hybrosomes were prebound with magnetic beads and subsequently labeled with fluorescent-dyed antibodies for the detection of exosome-specific CD9, CD63, and CD81 biomarker proteins. As seen in Figure 1D, the markers of exosome, liposome, and hybrosome were measured as 94.17%, 4.03%, and 69.19% for CD9; 90.14%, 2.36%, and 75.69% for CD63; 96.89%, 8.85%, and 79.60% for CD81, respectively (*P* ≤ .05).

### Cellular Uptake of Hybrosomes

**Figure 2.**
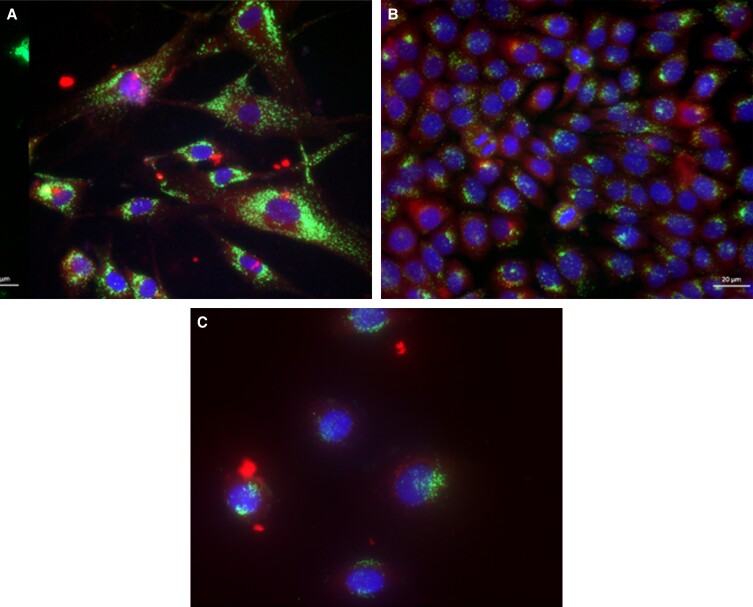
Hybrosome uptake of (A) HDF, (B) HaCaT, and (C) HUVEC cells (20× magnification, scale bar at 20 μm). HaCaT, human keratinocyte cell line; HDF, human dermal fibroblast cell line; HUVEC, human umbilical vein endothelial cells.

To investigate the internalization and cellular uptake performance of UCBP exosomes, liposomes, and hybrosomes, the cell nucleus and cell membrane of HDF, HUVEC, and HaCaT cells were stained with Hoechst and DAPI dyes, respectively. Moreover, UCBP exosome, liposome, and hybrosome staining were performed with lipophilic PKH67 fluorescent dye. The fluorescence intensities of PKH67 dyed in all 3 groups were quantified and standardized by cell number (DAPI count). Results of the experiment displayed that the uptake of hybrosomes into HDF, HUVEC, and HaCaT cells was high in cell lines after the treatment (Figure 2).

### Effects of Hybrosomes on Cell Proliferation

**Figure 3.**
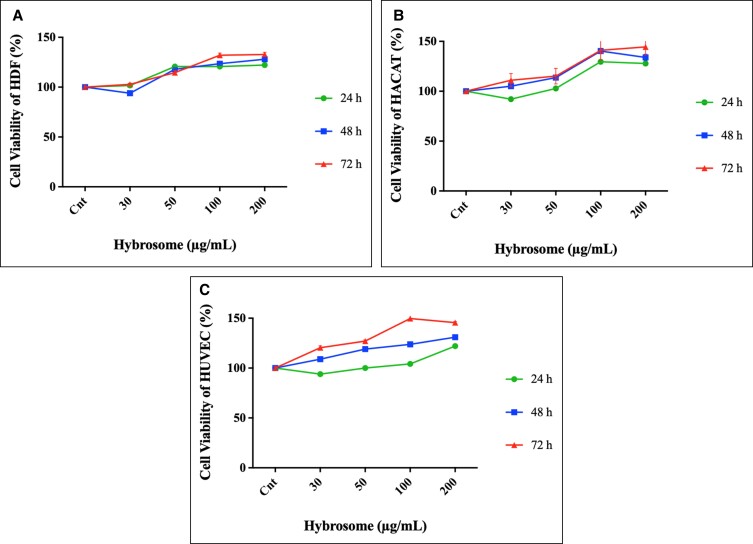
The cell viability of (A) HDF, (B) human HaCaT, and (C) HUVEC cells after the treatment of varying concentrations (30-200 μg/mL) of hybrosome after 24, 48, and 72 h incubation (37°C, 5% CO_2_) in DMEM supplemented with 10% FBS. Results represent the mean ± SD of 3 independent experiments (*P* ≤ .05). DMEM, Dulbecco's modified essential medium; FBS, fetal bovine serum; HaCaT, human keratinocyte cell line; HDF, human dermal fibroblast cell line; HUVEC, human umbilical vein endothelial cells; SD, standard deviation.

The cytotoxic effect of different concentrations of hybrosomes (30-50-100-200 μg/mL) on HDF, HUVEC, and HaCaT cells was determined within 3 days of the incubation (24, 48, and 72 h; Figure 3). HDF, HUVEC, and HaCaT cells that were not exposed to any of the hybrosomes were used as the control group for cell proliferation experiments. Cell viability of HDF cells were measured for control, 30, 50, 100, and 200 μg/mL groups after 48 h as 100.00 ± 1.57, 93.94 ± 1.01, 117.99 ± 1.58, 123.62 ± 1.64, and 128.03 ± 2.14; and after 72 h as 100.00 ± 2.07, 102.85 ± 1.00, 114.51 ± 2.21, 132.10 ± 2.07, and 132.81 ± 2.07, respectively. Cell viability of HUVEC cells were measured for control, 30, 50, 100, and 200 μg/mL groups after 48 h as 100.00 ± 1.21, 108.94 ± 1.96, 119.08 ± 1.64, 123.85 ± 1.21, and 130.88 ± 1.40; and after 72 h as 100.00 ± 1.42, 120.45 ± 1.84, 127.06 ± 1.15, 149.70 ± 1.07, and 145.51 ± 1.20, respectively. Cell viability of HaCaT cells were measured for control, 30, 50, 100, and 200 μg/mL groups after 48 h as 100.00 ± 0.20, 105.07 ± 0.80, 113.68 ± 0.70, 140.32 ± 2.60, and 133.95 ± 3.20; and after 72 h as 100.00 ± 0.10, 111.10 ± 6.70, 115.17 ± 7.80, 141.04 ± 9.70, and 144.48 ± 8.00, respectively. As a result, hybrosome concentrations up to 200 µg/mL did not show any cytotoxic effects on the 3 types of cell lines. Results demonstrated that cell proliferation was increased significantly in a time- and dose-dependent manner. Furthermore, as seen from the graphs of HUVEC and HaCaT cells, cell viability results at 200 µg/mL after 72 h showed decreasing curves compared to those at 100 µg/mL, while the cell viability at 200 and 100 µg/mL in HDF cells after 72 h was approximately same. In this circumstance, the optimum dose for maximum efficacy was determined to be 100 µg/mL. The percentage of cell proliferation was estimated by assigning 100% of the absorbance value obtained from the control cells. Results represent the mean ± SD of 3 independent experiments (*P* ≤ .05).

### The Activity of Hybrosomes on Cell Migration

In the scope of the cell proliferation assay, it is determined that 100 μg/mL hybrosome treatment increased the proliferation of dermal cells. In this circumstance, 100 μg/mL was chosen as the optimum dose for further hybrosome experiments. The activity of hybrosomes on cell migration functions was investigated through the scratch assay to determine the certain effect of hybrosomes on cell migration.

**Figure 4.**
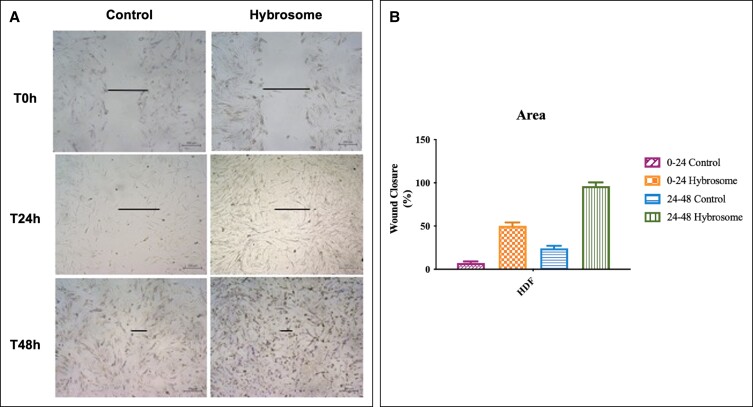
(A) Microscope images (Å ∼40×, 400 μm scale bar) to evaluate wound healing in vitro in the scratch assay using a confluent monolayer of HDF cells. (B) Cell migration was observed throughout the artificial wound immediately after injury and 24 and 48 h after incubation. Results represent the mean ± SD of 3 independent experiments in wound closure rate (*P* ≤ .05). HDF, human dermal fibroblast cell line; SD, standard deviation.

Control group was selected as HDF cells that had not been treated with hybrosomes, and both control and experimental groups, which included hybrosome supplied with HDF cells, were incubated at 37°C under 5% CO_2_. Although it was observed that the cells in the control group partially covered the scratched area after 48 h, the results of the scratch assay determined that hybrosome-treated HDF cells migrated more rapidly after 24 h incubation (Figure 4). Results represent the mean ± SD of 3 independent experiments in wound closure rate (*P* ≤ .05).

### Immunocytochemistry Analyses of Collagen Type I and Elastin Expression in Human Dermal Fibroblast Cells Treated With Hybrosomes

**Figure 5.**
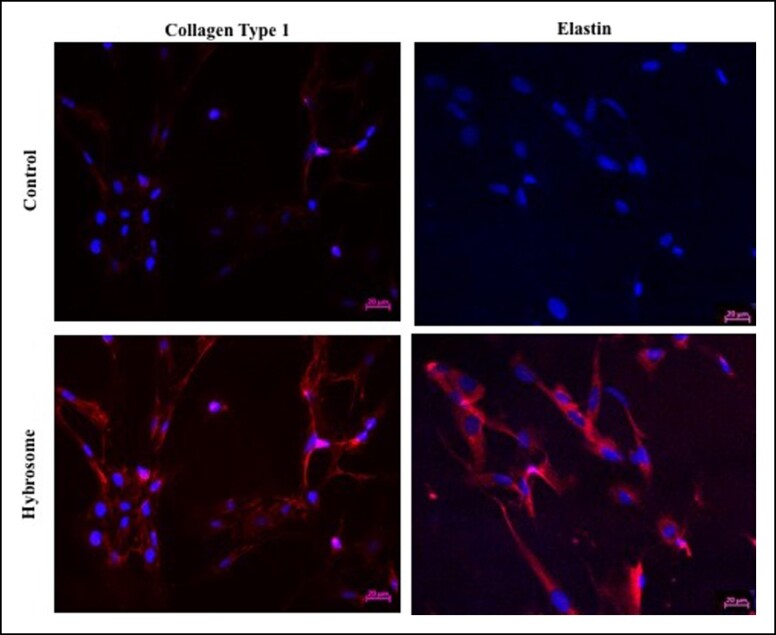
Confocal scanning microscope (20 μm scale bar, 20× magnification) image of the immunocytochemistry analysis of HDF cells to evaluate collagenase Type I and elastin expressions. Untreated control cells stain relatively weakly when stained with antibodies against collagen Type I and elastin than cells that have been treated with the hybrosome. DAPI-stained nuclei appear blue. Three independent experiments were repeated for immunocytochemistry studies and only 1 result image added for each group. DAPI, diamidino-2-phenylindole; HDF, human dermal fibroblast cell line.

Collagen Type 1 and elastin expression levels of untreated HDF control cells and hybrosome-treated HDF cells were investigated by immunocytochemistry analyses. Antibodies against collagen Type I and elastin were used to label the cells. According to the immunocytochemistry results, untreated control cells stain relatively weaker than hybrosome-treated HDF cells when dyed with antibodies against collagen Type I and elastin (Figure 5). Three independent experiments were repeated for immunocytochemistry studies and only 1 result image added for each group.

### Anti-inflammation Effect of Hybrosomes

**Figure 6.**
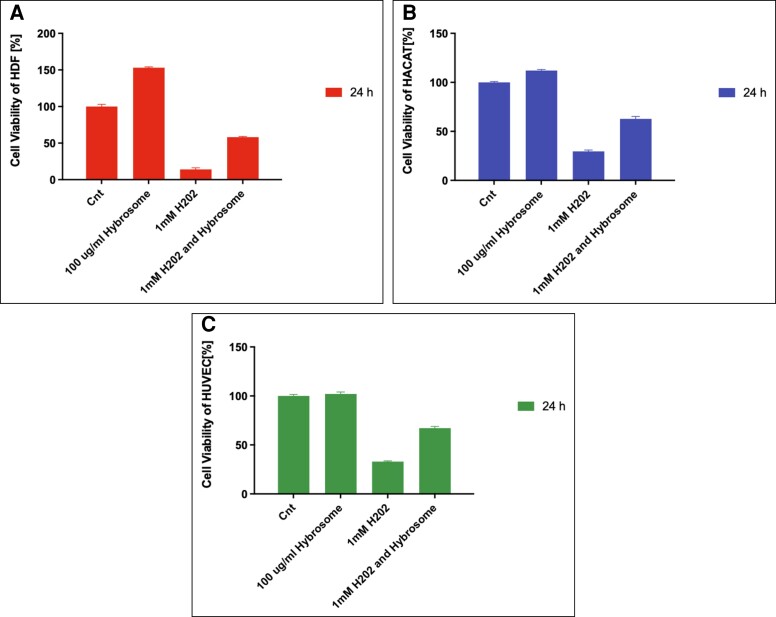
Anti-inflammation effect of hybrosome using H_2_O_2_ on (A) HDF, (B) HaCaT, and (C) HUVEC cell viability is obtained by taking 100 μg/mL hybrosome as positive control. Results represent the mean ± SD of 3 independent experiments (*P* ≤ .05). SD, standard deviation. HaCaT, human keratinocyte cell line; HDF, human dermal fibroblast cell line; HUVEC, human umbilical vein endothelial cells.

To determine the effect of hybrosomes under inflammatory conditions, untreated HDF, HUVEC, and HaCaT cells constituted the control group and were treated with normal culture mediums, while other groups were exposed to 100 μg/mL hybrosome or 1.0 mM hydrogen peroxide or both together. After 24 h of incubation, a cell proliferation assay was performed. Cell viability of HDF, HUVEC, and HaCaT cells was measured for control, 100 μg/mL hybrosome, 1 mM H_2_O_2_, and 1 mM H_2_O_2_ and hybrosome groups after 24 h as 100.00 ± 5.30, 153.00 ± 2.09, 14.00 ± 3.80, and 58.00 ± 1.93, respectively (Figure 6). Results represent the mean ± SD of 3 independent experiments (*P* ≤ .05).

### Gene Expression Analysis With Real-Time Polymerase Chain Reaction (RT-PCR)

**Figure 7.**
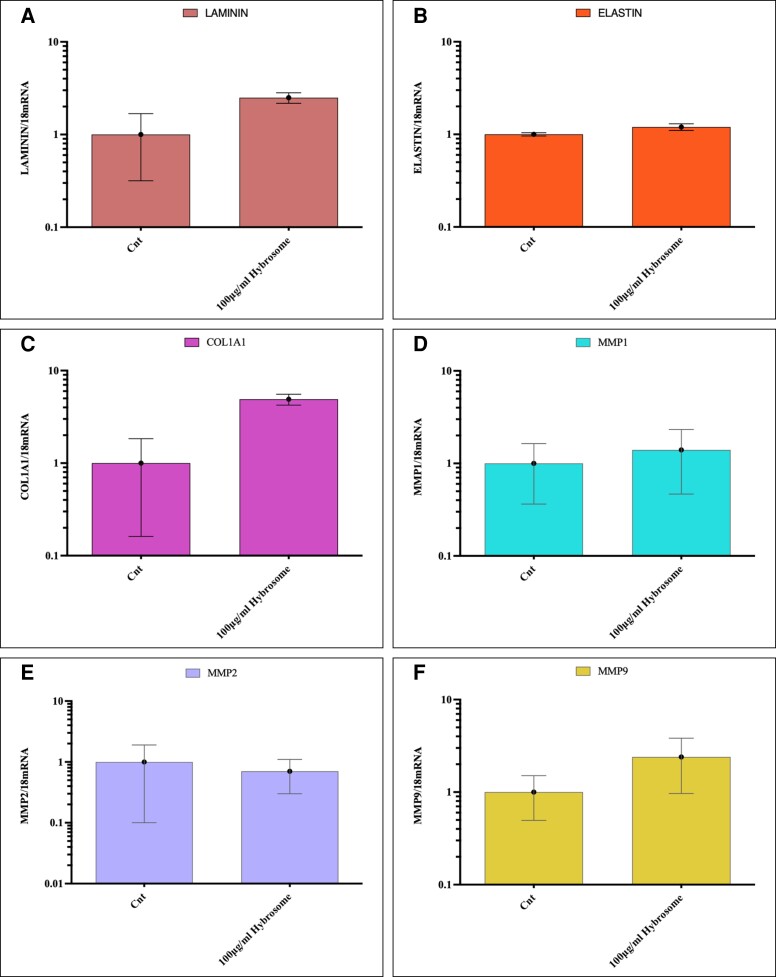
Wound healing related (A) laminin, (B) elastin, (C) COL1A1, (D) MMP1, (E) MMP2, and (F) MMP9 mRNA expression levels of HDF cells were evaluated using real-time polymerase chain reaction (RT-PCR) after the treatment of 100 μg/mL of hybrosome for 24 h. Both control groups and 100 μg/mL hybrosome-treated groups were incubated (37°C, 5% CO_2_) in DMEM medium supplemented with 10% FBS. Results represent 3 independent experiments average and mRNA levels were normalized against 18 mRNA expression levels. DMEM, Dulbecco's modified essential medium; FBS, fetal bovine serum; HDF, human dermal fibroblast cell line; mRNA, messenger ribonucleic acid; RNA, ribonucleic acid.

HDF cells were treated with 100 µg/mL hybrosome for 24 h to examine the potential effect of hybrosome on proliferation and migration activity and wound healing–related gene expression levels were assessed with RT-PCR analysis. As seen in Figure 7, gene expression levels of 100 μg/mL hybrosome-treated groups were measured as 4.90 ± 0.65 for COL1A1; 2.50 ± 0.33 for laminin; 1.20 ± 0.10 for elastin; 1.40 ± 0.93 for MMP1; 0.70 ± 0.40 for MMP2; 2.40 ± 1.43 for MMP9 (*P* ≤ .05). Results represent average and mRNA levels of control groups for each gene in 3 independent experiments which were normalized against 18s RNA expression levels.

### Enzyme-Linked Immunosorbent Assay

**Figure 8.**
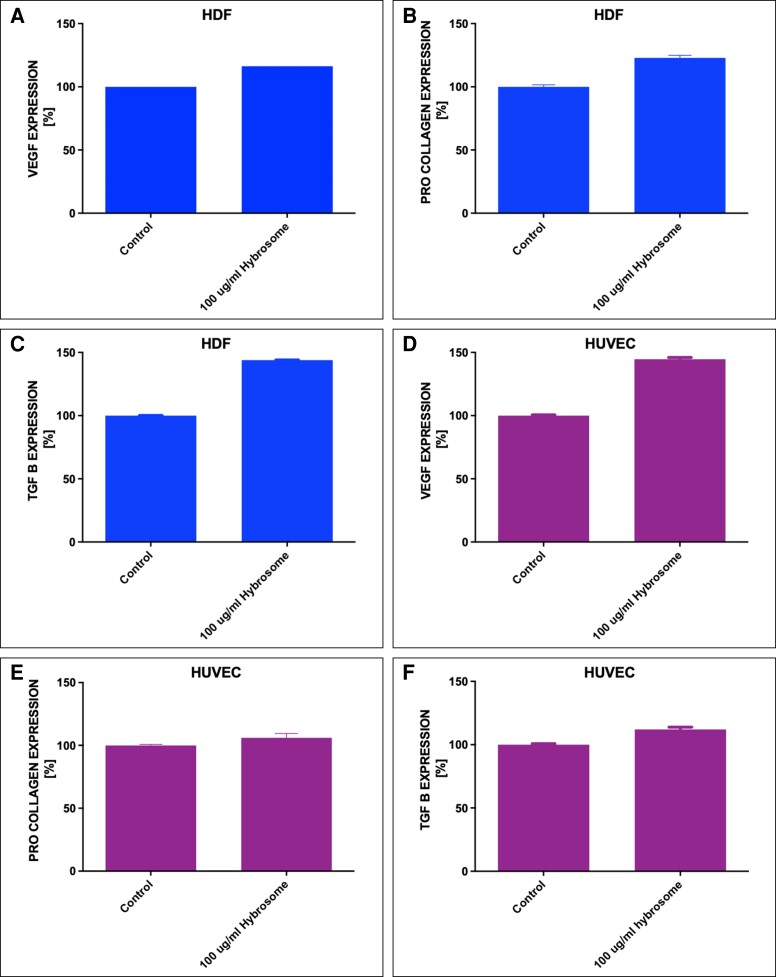
VEGF, human pro-collagen 1 alpha 1, and human TGF-beta 1 protein expression levels of (A, B, C) HDF and (D, E, F) HUVEC cells. Control was treated with only complete growth media. Each data set represents the analyses of 2-tailed multiple *t* tests, *n* = 3, *P < .05. HDF, human dermal fibroblast cell line; HUVEC, human umbilical vein endothelial cells; TGF, transforming growth factor; VEGF, vascular endothelial growth factor.

ELISA was conducted to investigate the protein expression levels of human VEGF, human pro-collagen 1 alpha 1, and human TGF-beta 1 in HDF and HUVEC cells after 100 μg/mL hybrosome application. Control group was supplied with only complete growth media. As seen in Figure 8, protein expression levels of 100 μg/mL hybrosome-treated HDF and HUVEC cells compared with control group. The HDF cell protein expression levels were measured as 116.28 ± 0.02 for VEGF; 122.90 ± 3.56 for pro-collagen 1 alpha 1; 143.95 ± 1.13 for TGF-beta 1. HUVEC cell protein expression levels of 100 μg/mL hybrosome groups were measured as 144.63 ± 2.40 for VEGF; 106.00 ± 6.00 for pro-collagen 1 alpha 1; 112.00 ± 3.23 for TGF-beta 1. Results were then analyzed by 2-tailed multiple *t* tests, *n* = 3, *P < .05.

## DISCUSSION

Wound healing is a complex biological process that consists of hemostasis, inflammation, proliferation, and tissue remodeling. The healing of tissue wounds requires a well-orchestrated integration of cell migration and proliferation, collagen synthesis and deposition, angiogenesis, and wound remodeling.^1,2^ This orderly dynamic process is provided by a series of intrinsic and extrinsic factors such as platelets, endothelial cells, inflammatory cells, cytokines, and growth factors which play an essential role.^1,3^

Plastic surgery and wound healing are closely related fields, as plastic surgery often involves repairing or reconstructing tissue that has been damaged by injury, disease, or surgery. Plastic surgery can help facilitate wound healing by providing additional support or coverage to the wound site. For example, skin grafts or tissue flaps can be used to cover large wounds or areas where the skin has been lost. These techniques can help to speed up the healing process and reduce the risk of infection. In addition to aiding wound healing, plastic surgery can also improve the cosmetic appearance of scars. Scar revisional surgery can help to minimize the appearance of scars by reducing their size or altering their shape. This can be especially important for patients who have scars in visible areas of the body, such as the face or hands.^24,25^

Recent studies have revealed that body fluids, plasma applications, and biological nanovesicles for wound healing continue to attract great interest in plastic surgery and regenerative medicine. Exosomes are nano-sized membrane-derived vesicles that are known as significant paracrine factors and play a crucial role in mediating intercellular communication. They are acquired from bodily fluids, including plasma, and are secreted by the majority of cells.^14^ In plastic surgery, different types of exosome treatment approaches are continuing to try, including the promotion of wound healing and tissue regeneration.^26,27^

Umbilical cord blood is an essential source of exosomes and is rich in stem cells. UCBP possesses further distinct advantages when compared with other sources. They are safer for donors, easier to access, and cause a lower incidence of graft-vs-host disease thus making them an attractive source of stem cell transplantation. It has been established that UCBP promotes angiogenesis and speeds up wound healing. Recent studies indicate that exosomes from UCBP result in accelerated re-epithelialization, reduced scar widths, and all together facilitated wound repair and regeneration.^13,14^ Thanks to these properties of umbilical cord blood exosomes, in plastic surgery, cord blood-derived exosomes may have a variety of clinical applications, including promoting wound healing and tissue regeneration after surgical procedures such as breast reconstruction or skin grafting.

In the literature, there are various animal and human-sourced umbilical cord blood studies.^13,14^ However, since exosomes are messenger molecules that carry the content of their origin, obtaining approval from the authorities for the therapeutic usage of human-derived exosomes and proving that the content of the source is safe can be challenging. Additionally, specific protocols must be followed to obtain human tissue, and these protocols are subject to tight regulations. For this reason, obtaining exosomes from newborn animal tissue not only facilitates large-scale production but also makes it possible to obtain a purer content.

The hybrid exosome–liposome system, a type of artificial vesicle that combines the properties of both exosomes and liposomes, is promising for the development of new applications in regenerative medicine due to its advanced properties. The hybrid exosome–liposome structures are created by fusing exosomes with liposomes, resulting in a structure that retains the surface properties of exosomes while also having the ability to encapsulate and deliver cargo-like liposomes.^18^ This hybrid structure can potentially enhance the stability and specificity of the cargo delivery process while also providing a natural “fingerprint” that can help the vesicles to target specific cells or tissues. In the context of wound healing, hybrid exosomes can be designed to contain molecules that promote cell growth, modulate inflammation, and enhance tissue regeneration.

In this study, we developed a new hybrid liposome–exosome technology called “hybrosome” to combine the enhanced delivery capacity of liposomes with targeting properties of exosomes derived from UCBP to improve wound healing. We demonstrated in vitro that UCBP exosomes have high cellular uptake and migration capacity in fibroblast, endothelium, and keratinocyte cell lines, as well as increase the expression of precursor proteins such as collagen and elastin, which are markers of the wound-healing process, at a higher rate compared to control groups, as supported by immunocytochemistry, ELISA, and RT-PCR results. Our results strongly suggest that UCBP exosomes may be delivered into localized keratinocyte, fibroblast, and endothelial cells to stimulate their regenerative responses, speeding up wound healing and regeneration. According to the outputs, we demonstrated that hybrosome can be used as a cutting-edge therapeutic nano-delivery method for the treatment of wounds.

To be able to accurately identify the hybrosome molecule, both the final product and its main components, the UCBP exosome, and liposome, need to be correctly characterized and the results compared with each other. For this reason, UCBP exosome, hybrosome, and liposome were used as 3 different groups separately and characterized according to the MISEV20 standard. As demonstrated in Figure 1, the isolation of homogeneous and stable exosome-like nanoparticles from UCBP was effective. Isolated nanoparticles were evaluated through NTA assay and flow cytometry to characterize their morphology, size distribution, and concentration.

NTA observes each particle moving through a Brownian motion and determines its size by analyzing how each particle spreads within the dispersant. As determined by NTA analysis, the UCBP exosome, liposome, and hybrosome had size ranges of 106, 191, and 139 nm, respectively. These findings are consistent with previous studies and demonstrate that nanovesicles isolated from blood plasma are exosome-like nanoparticles.^28^ Furthermore, prepared egg yolk lecithin liposomes are large, unilamellar vesicles (SUV; 100-200 nm). Following the NTA findings, it can be deduced that the hybridization stage was carried out successfully since the sizes of the hybrosome vary between exosome and liposome.

The percentages of exosome-specific CD markers on the surfaces of the exosome, liposome, and hybrosome structures were determined by using an advanced type of flow cytometry called fluorescence-activated cell sorting. To detect specific CD markers, magnetic beads were bound with exosomes, liposomes, and hybrosomes, which were subsequently fluorescently labeled for further flow cytometric analysis. Exosomes, liposomes, and hybrosomes stained with the exosome-specific tetraspanin markers known as CD9, CD63, and CD81 were examined as common CD surface indicators for the antibody-stained sample groups due to their widespread distribution in a variety of tissues. All exosome-specific markers resulted in a high percentage rate for exosomes and a low percentage rate for liposomes. Hybrosome CD surface markers percentage varies between exosome and liposome.

In acute wounds, the proliferative phase starts after the inflammatory process. To bridge the gap, this stage involves both cell migration and proliferation steps together.^2–4^ This is a rate-limiting healing factor that must be presented for effective healing. The application of hybrosomes to the cell lines evaluated in this work resulted in a considerable increase in cell proliferation (40%-50%) in a dosage-dependent manner, based on our findings from the MTS assays. To assess the cytotoxicity of hybrosomes and their impact on cell proliferation, the MTS test was used for 3 days, monitoring cell viability at 24, 48, and 72 h time points at 4 different doses ranging from 30 to 200 g/mL in the HDF, HACAT, and HUVEC cell lines. As shown in Figure 2, no cellular toxicity was identified at any of the doses after hybrosome treatment. All dosages have been found to significantly increase cell viability and thus cell number. On the other hand, after analyzing the 3-day data, it was understood that the most significant increase was obtained as a result of 100 μg/mL treatment, and it was concluded that this would be the best hybrosome application dosage for future studies.

Fibroblast cells are the primary effectors for soft-tissue wound healing and their migration is necessary for wound contraction, collagen formation, and tissue remodeling.^29^ In here, we assessed how hybrosomes affected the behavior of fibroblasts in vitro. Migration is also known as a rate-limiting aspect of the wound-healing process in terms of inflammation prevention and immune response activation. The scratch assay is widely considered the gold standard for determining wound closure rate. A scratch assay was performed to demonstrate the potential effect of hybrosomes on cell migration. In this assay, a gap formed by scratching the plate is tracked over time to determine the efficacy of gap closure in terms of cell migratory patterns, which is used to represent wound closure. The scratch assay revealed that, while control group cells modestly closed the scratched area after 48 h, HDF cells treated with hybrosomes migrated faster, resulting in a 50% higher wound closure rate after 24 h therewithal migration is 4 times higher in HDF cells treated with hybrosome compared to the control group at the end of 48 h. According to these results, it was observed that hybrosomes have a crucial role in cell migration by promoting wound closure. The findings indicated that these nanoparticles could be internalized by fibroblasts and dramatically increased their proliferation and migration potential, demonstrating that the activation of fibroblasts is a way through which hybrosomes facilitate wound healing.

Anti-inflammation is a major process in the wound-healing process. Hydrogen peroxide (H_2_O_2_) tests are used in vitro research to drive the onset and simulate inflammation, through the activation of nuclear factor kappa B transcription factors.^30^ H_2_O_2_ was utilized to generate oxidative stress on HDF, HUVEC, and HACAT cells to test their anti-inflammatory abilities. Hydrogen peroxide anti-inflammation assay results revealed that 1 mM H_2_O_2_ application had a cytotoxic effect on cell lines and cell viability of this group was lower compared to other healthy cells. It was observed that the cell viability results of both hybrosome and H_2_O_2_-treated groups were close to the positive group with only hybrosome. As seen in Figure 6 compared to the group that received only H_2_O_2_ treatment, cell viability in HDF cells treated with H_2_O_2_ and hybrosome increased by a 4.14-fold, in HUVEC cells by a 2.034-fold, and in HaCaT cells by a 2.112-fold. This data indicated that the hybrosome reduced the cytotoxic effect of H_2_O_2_. Therefore, it was determined that the hybrosome had an anti-inflammatory effect on HDF, HUVEC, and HaCaT cells. It is concluded here that hybrosomes can shield fibroblast cells against inflammation caused by reactive oxygen species.

Collagen and elastin are extracellular matrix components that play a vital role in wound healing.^31^ The wound-healing process necessitates the replacement of previously formed fibrin clots with collagen matrix Type I. Collagen, which is produced by fibroblasts, regulates cell adhesion and migration during skin healing.^32^ As a result, our findings substantially support the hypothesis that hybrosomes increase collagen and elastin expression levels based on immunocytochemistry data as shown in Figure 5. RT-PCR results showed that hybrosome-treated HDF cells resulted in around a 4.9-fold increase in COL1A1, a 2.5-fold increase in laminin, a 1.4-fold increase in MMP1, and a 2.4-fold increase in MMP9 mRNA expression levels, respectively. On the other hand, there was no significant change detected in MMP2 and elastin gene expression levels. Accordingly, increased mRNA levels of collagen Type I and laminin resulted in collagen formation as well as increased fibroblast cell proliferation and migration throughout the wound-healing process. MMP-1, MMP-2, and MMP9 break down temporary wound matrix and extracellular matrix proteins to create an ideal environment for cell migration.^33^ The cells treated with hybrosomes showed an increase in MMPs, which play a role in angiogenesis which is a critical stage of wound healing. Although the precise function of TGF proteins in wound healing is unknown, it has been reported that TGF-1 and TGF-3 increase fibroblast and keratinocyte cell migration and proliferation, granulation tissue formation, extracellular matrix development, growth factor secretion production, angiogenesis, tissue remodeling.^34^ ELISA results demonstrated that hybrosome-treated HDF cells resulted in a 22% increase in COL1A1, a 43% increase in TGF-beta 1, and a 16% increase in VEGF protein expression levels, respectively. Moreover, hybrosome-treated HUVEC cells also resulted in a 6% increase in COL1A1, a 12% increase in TGF-beta 1 protein expression levels, and a 44% increase in VEGF protein expression levels, respectively. According to our findings, fibroblast and keratinocyte cell TGF-beta expression levels increased in response to hybrosome administration. Furthermore, after hybrosome treatment of HDF and HUVEC cells, VEGF, also known as a VEGF, which is vital in vascularization and wound healing, was dramatically upregulated.

### Limitations of the Study

This present study has been subject to a variety of limitations. Firstly, in this study, scratch assays and wound healing–specific gene expression analyses were examined to determine the effect of hybrosome technology on wound healing. To support this study, our advanced in vitro studies are continuing and will be published in the future. Secondly, it is currently unknown whether prolonged delivery of hybrosomes can provide comparable or much better pro-regenerative effects in mouse skin wounds. Therefore, in vivo animal studies should be performed to examine the regenerative effect of the hybrosome and strongly support the in vivo experimental results. Moreover, miRNA profiling and proteomic studies should be conducted in future studies to investigate the specific molecular mechanism behind the regenerative effect of hybrosomes and the regulated target gene expressions. Lastly, in the present study, exosome, liposome, and hybrosome groups were compared with each other just in a characteristic manner in vitro studies. In future studies, a comparison of these 3 groups may be conducted for other researchers to analyze these hybrosome efficiencies more accurately.

## CONCLUSIONS

Although extracellular vesicle-based experiments have recently exploded in exosome–liposome-based research, their effect on wound healing has yet to be investigated. In this study, a new approach named hybrosome was developed by hybridizing calf UCBP-derived exosomes and liposomes for the first time. Our experimental results strongly show that hybrosomes had a great ability in tissue wound healing using in vitro approaches. Considering these observations, hybrosome technology is a promising new-generation bioengineering approach in the field of regenerative medicine, and further studies should be conducted in detail to observe its regeneration potential and wound-healing capabilities.

## References

[1] Broughton G 2nd , Janis JE, Attinger CE. The basic science of wound healing. Plast Reconstr Surg. 2006;117(7 Suppl):12S–34S. doi: 10.1097/01.prs.0000225430.42531.c2 16799372

[2] Braiman-Wiksman L , Solomonik I, Spira R, Tennenbaum T. Novel insights into wound healing sequence of events. Toxicol Pathol. 2007;35(6):767–779. doi: 10.1080/01926230701584189 17943650

[3] Hosgood G . Stages of wound healing and their clinical relevance. Vet Clin North Am Small Anim Pract. 2006;36(4):667–685. doi: 10.1016/j.cvsm.2006.02.006 16787782

[4] Wilkinson HN , Hardman MJ. Wound healing: cellular mechanisms and pathological outcomes. Open Biol. 2020;10(9):200223. doi: 10.1098/rsob.200223 PMC7536089 32993416

[5] Li J , Chen J, Kirsner R. Pathophysiology of acute wound healing. Clin Dermatol. 2007;25(1):9–18. doi: 10.1016/j.clindermatol.2006.09.007 17276196

[6] Camussi G , Deregibus MC, Bruno S, Cantaluppi V, Biancone L. Exosomes/microvesicles as a mechanism of cell-to-cell communication. Kidney Int. 2010;78(9):838–848. doi: 10.1038/ki.2010.278 20703216

[7] Şahin F , Koçak P, Güneş MY, Özkan İ, Yıldırım E, Kala EY. In vitro wound healing activity of wheat-derived nanovesicles. Appl Biochem Biotechnol. 2019;188(2):381–394. doi: 10.1007/s12010-018-2913-1 30474796

[8] Kahlert C , Kalluri R. Exosomes in tumor microenvironment influence cancer progression and metastasis. J Mol Med (Berl). 2013;91(4):431–437. doi: 10.1007/s00109-013-1020-6 23519402 PMC4073669

[9] An Q , Hücvkelhoven R, Kogel K, Handvan Bel AJ. Multi vesicular bodies participate in a cell wall-associated defence response in barley leaves attacked by the pathogenic powdery mildew fungus. Cell Microbiol. 2006;8(6):1009–1091. doi: 10.1111/j.1462-5822.2006.00683.x 16681841

[10] Ocansey DKW , Zhang L, Wang Y, et al Exosome-mediated effects and applications in inflammatory bowel disease. Biol Rev Camb Philos Soc. 2020;95(5):1287–1307. doi: 10.1111/brv.12608 32410383 PMC7540363

[11] Gogolak P , Rethi B, Hajas G, Rajnavolgyi E. Targeting dendritic cells for priming cellular immuneresponses. J Mol Recognit. 2003;16(5):299–317. doi: 10.1002/jmr.650 14523943

[12] Elkhoury K , Koçak P, Kang A, Arab-Tehrany E, Ellis Ward J, Shin SR. Engineering smart targeting nanovesicles and their combination with hydrogels for controlled drug delivery. Pharmaceutics. 2020;12(9):849. doi: 10.3390/pharmaceutics12090849 32906833 PMC7559099

[13] Hu Y , Rao S-S, Wang Z-X, et al Exosomes from human umbilical cord blood accelerate cutaneous wound healing through miR-21-3p-mediated promotion of angiogenesis and fibroblast function. Theranostics. 2018;8(1):169–184. doi: 10.7150/thno.21234 29290800 PMC5743467

[14] Zhang J , Chen C, Hu B, et al Exosomes derived from human endothelial progenitor cells accelerate cutaneous wound healing by promoting angiogenesis through Erk1/2 signaling. Int J Biol Sci. 2016;12(12):1472–1487. doi: 10.7150/ijbs.15514 27994512 PMC5166489

[15] Zhang M , Viennois E, Xu C, Merlin D. Plant derived edible nanoparticles as a new therapeutic approach against diseases. Tissue Barriers. 2016;4(2):e1134415. doi: 10.1080/21688370.2015.1134415 PMC4910829 27358751

[16] Šturm L , Poklar Ulrih N. Basic methods for preparation of liposomes and studying their interactions with different compounds, with the emphasis on polyphenols. Int J Mol Sci. 2021;22(12):6547. doi: 10.3390/ijms22126547 34207189 PMC8234105

[17] Akbarzadeh A , Rezaei-Sadabady R, Davaran S, et al Liposome: classification, preparation, and applications. Nanoscale Res Lett. 2013;8(1):102. doi: 10.1186/1556-276X-8-102 23432972 PMC3599573

[18] Sato YT , Umezaki K, Sawada S, et al Engineering hybrid exosomes by membrane fusion with liposomes. Sci Rep. 2016;6(1):21933. doi: 10.1038/srep21933 26911358 PMC4766490

[19] Hannafon BN , Ding W-Q. Intercellular communication by exosome-derived microRNAs in cancer. Int J Mol Sci. 2013;14(7):14240–14269. doi: 10.3390/ijms140714240 23839094 PMC3742242

[20] Sun L , Fan M, Huang D, et al Clodronate-loaded liposomal and fibroblast-derived exosomal hybrid system for enhanced drug delivery to pulmonary fibrosis. Biomaterials. 2021;271:120761. doi: 10.1016/j.biomaterials.2021.120761 33774524

[21] Lin Y , Wu J, Gu W, et al Exosome-liposome hybrid nanoparticles deliver CRISPR/Cas9 system in MSCs. Adv Sci (Weinh). 2018;5(4):1700611. doi: 10.1002/advs.201700611 PMC5908366 29721412

[22] Antimisiaris SG , Mourtas S, Marazioti A. Exosomes and exosome-inspired vesicles for targeted drug delivery. Pharmaceutics. 2018;10(4):218. doi: 10.3390/pharmaceutics10040218 30404188 PMC6321407

[23] Zhao H , Li Z, Wang Y, et al Bioengineered MSC-derived exosomes in skin wound repair and regeneration. Front Cell Dev Biol. 2023;11:1029671. doi: 10.3389/fcell.2023.1029671 PMC10009159 36923255

[24] Niederstätter IM , Schiefer JL, Fuchs PC. Surgical strategies to promote cutaneous healing. Med Sci (Basel). 2021;9(2):45. doi: 10.3390/medsci9020045 34208722 PMC8293365

[25] Nauta A , Gurtner G, Longaker MT. Wound healing and regenerative strategies. Oral Dis. 2011;17(6):541–549. doi: 10.1111/j.1601-0825.2011.01787.x 21332599

[26] Zhang B , Gong J, He L, et al Exosomes based advancements for application in medical aesthetics. Front Bioeng Biotechnol. 2022;10:1083640. doi: 10.3389/fbioe.2022.1083640 PMC9810265 36605254

[27] Safari B , Aghazadeh M, Davaran S, Roshangar L. Exosome-loaded hydrogels: a new cell-free therapeutic approach for skin regeneration. Eur J Pharm Biopharm. 2022;171:50–59. doi: 10.1016/j.ejpb.2021.11.002 34793943

[28] Brennan K , Martin K, FitzGerald SP, et al A comparison of methods for the isolation and separation of extracellular vesicles from protein and lipid particles in human serum. Sci Rep. 2020;10(1):1039. doi: 10.1038/s41598-020-57497-7 31974468 PMC6978318

[29] Driskell RR , Lichtenberger BM, Hoste E, et al Distinct fibroblast lineages determine dermal architecture in skin development and repair. Nature. 2013;504(7479):277–281. doi: 10.1038/nature12783 24336287 PMC3868929

[30] Lisanti MP , Martinez-Outschoorn UE, Lin Z, et al Hydrogen peroxide fuels aging, inflammation, cancer metabolism and metastasis: the seed and soil also needs “fertilizer”. Cell Cycle. 2011;10(15):2440–2449. doi: 10.4161/cc.10.15.16870 21734470 PMC3180186

[31] Potekaev NN , Borzykh OB, Medvedev GV, et al The role of extracellular matrix in skin wound healing. J Clin Med. 2021;10(24):5947. doi: 10.3390/jcm10245947 34945243 PMC8706213

[32] Velnar T , Bailey T, Smrkolj V. The wound healing process: an overview of the cellular and molecular mechanisms. J Int Med Res. 2009;37(5):1528–1542. doi: 10.1177/147323000903700531 19930861

[33] Caley MP , Martins VL, O'Toole EA. Metalloproteinases and wound healing. Adv Wound Care (New Rochelle). 2015;4(4):225–234. doi: 10.1089/wound.2014.0581 25945285 PMC4397992

[34] Lichtman MK , Otero-Vinas M, Falanga V. Transforming growth factor beta (TGF-β) isoforms in wound healing and fibrosis. Wound Repair Regen. 2016;24(2):215–222. doi: 10.1111/wrr.12398 26704519

