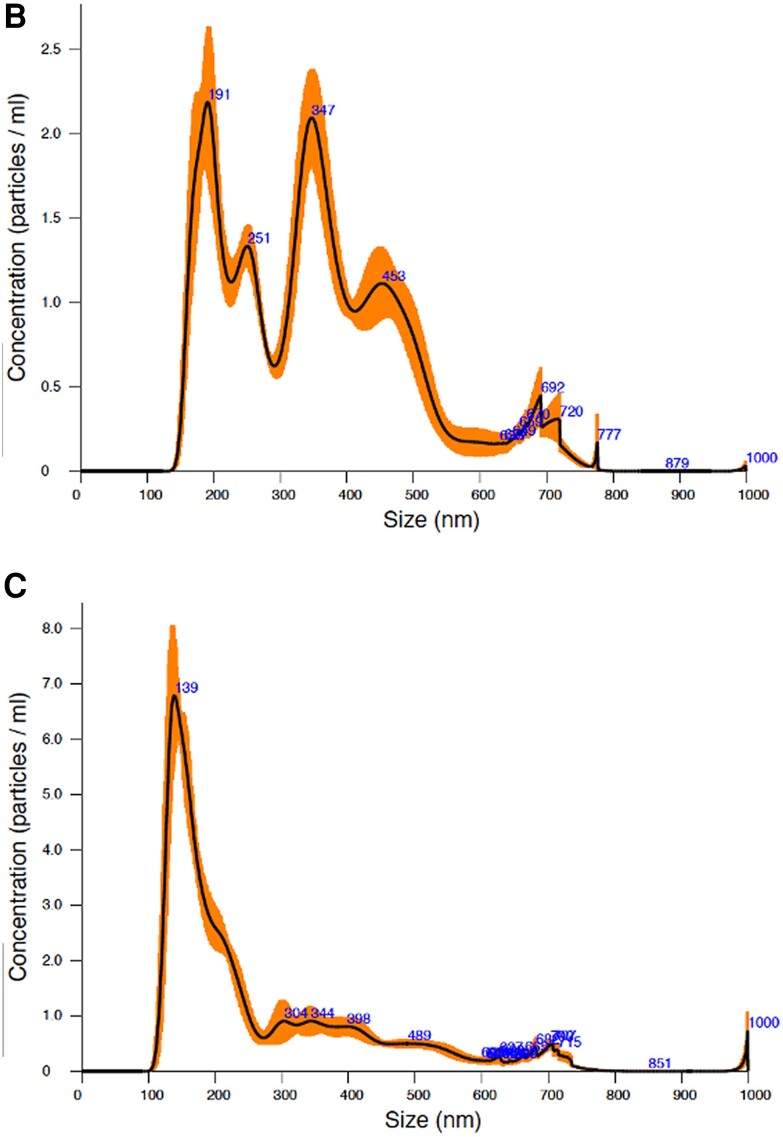# Correction to: The Effect of Hybrosome (Umbilical Cord Blood Exosome–Liposome Hybrid Vesicles) on Human Dermal Cells In Vitro

**DOI:** 10.1093/asjof/ojad086

**Published:** 2023-09-21

This is a correction to: Polen Koçak, Naz Unsal, Serli Canikyan, Yaren Kul, Steven R Cohen, Tunç Tiryaki, Diane Duncan, Kai-Uwe Schlaudraff, Benjamin Ascher, Teodor Eren Tiryaki, The Effect of Hybrosome (Umbilical Cord Blood Exosome–Liposome Hybrid Vesicles) on Human Dermal Cells In Vitro, *Aesthetic Surgery Journal Open Forum*, Volume 5, 2023, ojad039, https://doi.org/10.1093/asjof/ojad039

In the originally published version of this manuscript, the author regrets that there were errors in the original version of figure 1. The corrected versions of the figure parts are included here, and have been corrected online in the version of record.

**Figure 1:**